# Neo-Commissural Alignment by Withdrawing and Readvancing the Delivery System during Transcatheter Aortic Valve Replacement with Self-Expanding Prosthesis

**DOI:** 10.1155/2023/1060481

**Published:** 2023-12-11

**Authors:** Xian Liu, Yingdong Wang, Yuhe Sheng, Yaling Han, Quanmin Jing, Geng Wang, Zhenyang Liang, Yang Li, Bin Wang, Kai Xu, Li Yang, Gary S. Mintz

**Affiliations:** ^1^Department of Cardiology, General Hospital of Northern Theater Command, Shenyang 110016, China; ^2^National Engineering Research Center for Biomaterials, College of Biomedical Engineering, Sichuan University, Chengdu 610064, China; ^3^Cardiovascular Research Foundation, New York, NY, USA

## Abstract

**Objective:**

To investigate the feasibility of obtaining neo-commissural alignment by withdrawing and readvancing the delivery system during transcatheter aortic valve replacement (TAVR) with self-expanding prosthesis.

**Methods:**

TAVR was performed in five patients with severe aortic valve stenosis by the femoral approach. The delivery catheter was withdrawn and readvanced with the opposite orientation when the Venus-A plus transcatheter heart valve (THV) centre marker was found to be overlapped with or close to the left marker at the aortic annulus level on the fluoroscopic image at the projection of the right and left coronary cusps superimposing. Neo-commissural alignment was evaluated by comparing the aortic computed tomography before TAVR with it after TAVR.

**Results:**

The THV centre marker was overlapped with or close to the right marker at the aortic annulus level on the fluoroscopic image at the projection of the right and left coronary cusps superimposed in all the present five patients after withdrawing and readvancing the delivery system. The commissural angle deviation before vs. post TAVR was 12.3° ± 7.0°. Three of five patients had neo-commissural alignment. Two of the five patients had mild neo-commissural misalignment.

**Conclusions:**

It is possible to obtain the neo-commissural alignment by controlling delivery catheter insertion orientation using the markers on the inflow of the Venus-A plus valve.

## 1. Introduction

**Figure 1:**
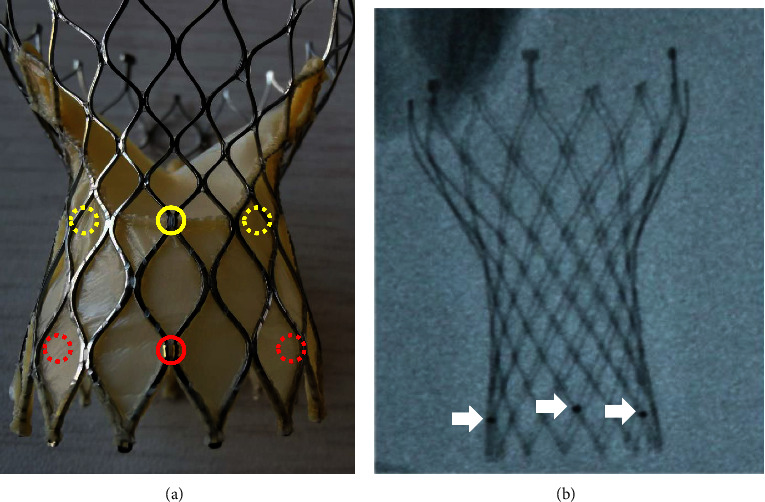
(a) Three markers (red circles) on the inflow of the THV are in alignment with the nadirs of the THV's sinuses (yellow circles). The circles with dotted lines indicate that they are at the back of the THV. (b) The markers can be seen under an X-ray (white arrows). THV = transcatheter heart valve.

If percutaneous coronary intervention is required after transcatheter aortic valve replacement (TAVR), coronary access will be more challenging when transcatheter heart valves (THVs) commissural posts face a coronary orifice, particularly with self-expanding valves [1]. Venus-A plus THV (Venus Medtech Inc, Hangzhou, China) has three inflow markers that are in alignment with the nadirs of THV sinuses (Figure 1) [2, 3]. Our previous study showed that neo-commissural alignment could be evaluated by observing the three markers of the Venus-A plus THV (3). However, it is not known whether neo-commissural alignment can be obtained by controlling the delivery system. We present a series of cases in which we obtained neo-commissural alignment by manipulating the delivery catheter insertion orientation relative to the three markers of the Venus-A plus THV [4].

## 2. Material and Methods

### 2.1. Patient Population and Delivery System Management

**Figure 2:**
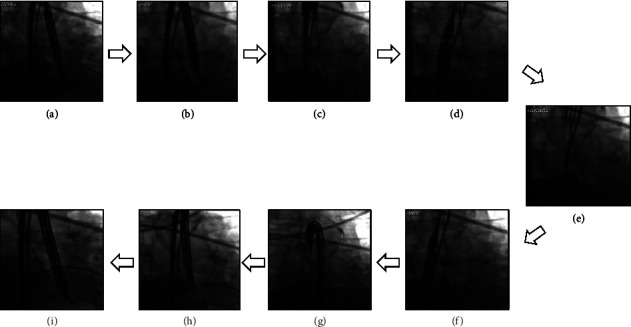
The procedure of withdrawing the delivery system to the descending aorta and readvancing the transcatheter heart valve to the aortic annulus was shown from panel a to panel i from case 1.

TAVR was performed in five patients with severe tricuspid or type 1 bicuspid aortic valve stenosis by the femoral approach [5]. The delivery catheter carrying the Venus-A plus THV was inserted into the femoral sheath with the flushing port at 9 o'clock, according to the delivery system manual. The THV centre marker was found to be overlapped with or close to the left marker at the aortic annulus level on the fluoroscopic image in the projection of the right and left coronary cusps superimposed at the first advancing of the delivery system in these five patients. The delivery catheter was withdrawn until the THV reached the descending aorta. The flushing port was positioned at 3 o'clock by rotating the delivery catheter 180°. The THV was then readvanced to the aortic annulus (Figure 2). Given that this was a pilot imaging study, our study was approved by the respective institutional review boards, and the requirement to obtain patient consent was waived.

### 2.2. Neo-Commissural Alignment Evaluation and Definition

Neo-commissural alignment was evaluated by comparing the aortic root computed tomography (CT) of 75% phase before TAVR with it after TAVR. The FluoroCT software developed by Pascal Thériault-Lauzier and Nicolo Piazza was used for CT analysis. Neo-commissural alignment was defined when the commissural angle deviation was 0° to 15°; mild, moderate, or severe neo-commissural misalignments were defined when the angle deviation was 15.1° to 30°, 30.1° to 45°, or 45.1° to 60°, respectively [6].

### 2.3. Statistical Analysis

Variables were presented as the mean ± standard deviation.

## 3. Results

### 3.1. Baseline Patient Characteristics

The classification system from Sievers and Schmidtke was used for describing the aortic valve in our study [5]. Baseline patient characteristics are shown in Table 1.

### 3.2. Neo-Commissure Alignment

#### 3.2.1. TAVR Procedure and Complications and CT Images

**Figure 3:**
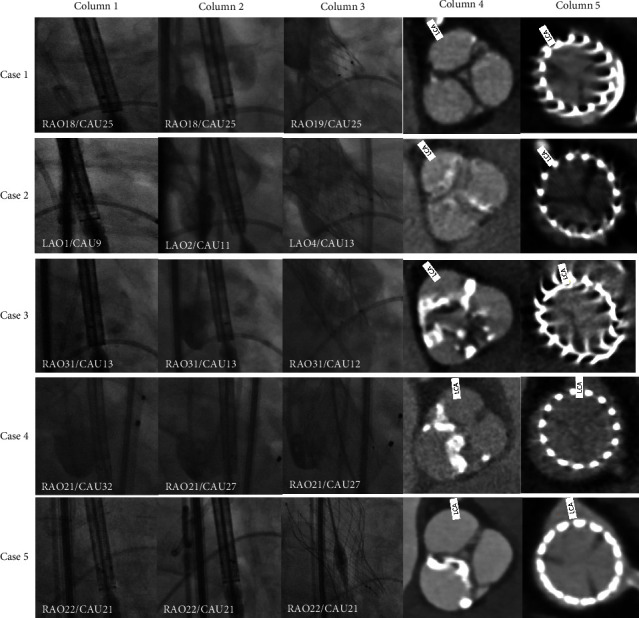
Neo-commissure alignment by manipulating the delivery system. Column 1: angiograms when the THV crossed the aortic annulus at the first time. The centre markers were close to or overlapped with the left markers. Column 2: angiograms after the delivery system were withdrawn to the descending aorta and readvanced to the aortic annulus. The centre markers were close to or overlapped with the right markers. Column 3: angiograms after the THV were deployed. Column 4: transverse aortic sinus CT images before TAVR. Column 5: transverse aortic sinus CT images after TAVR. THV = transcatheter heart valve; CT = computed tomography; TAVR = transcatheter aortic valve replacement; RAO = right anterior oblique; LAO = left anterior oblique; CAU = caudal; and LCA = left coronary artery.

The centre marker of THV was overlapped or close with the right marker at the aortic annulus on the fluoroscopic image at the projection of the right and left coronary cusps superimposing after the delivery system was withdrawn to the descending aorta and readvanced in all five cases without complications including cerebral infarction or vascular injury. Details are shown in Figure 3.

#### 3.2.2. Commissural Angle Deviation before vs. post TAVR

**Figure 4:**
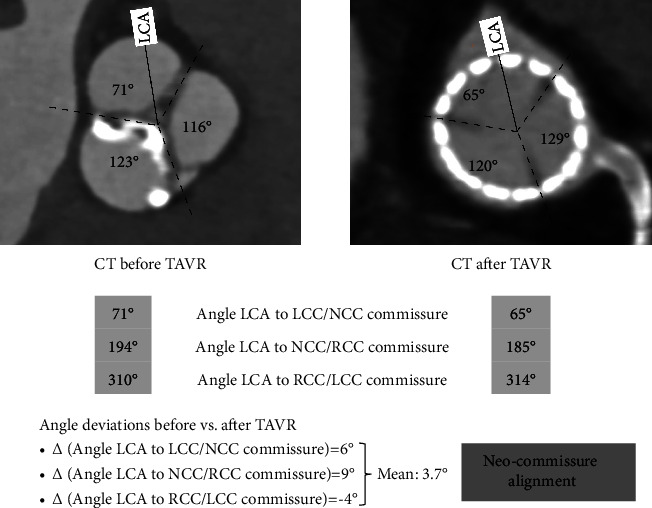
The procedure of obtaining the commissural angel deviation from CT before vs. after TAVR from case 5. LCA = left coronary artery; CT = computed tomography; TAVR = transcatheter aortic valve replacement; LCC = left coronary cusp; NCC = noncoronary cusp; RCC = right coronary cusp.

The commissural angle deviation before vs. post TAVR was 12.3° ± 7.0°. Three of 5 patients had neo-commissural alignment. Two of 5 had mild neo-commissural misalignment (Table 2). The example of obtaining the angle deviation is shown in Figure 4 from case 5.

## 4. Discussion

THV should be deployed with neo-commissural alignment in order to minimize difficulties for future coronary access. The success rate of TAVR with neo-commissural alignment will be high if the delivery system is inserted with the flushing port positioned at 3 o'clock (Evolut, Medtronic), 6 o'clock (ACURATE, Boston Scientific), or 12 o'clock (Portico, Abbott) [7].

**Figure 5:**
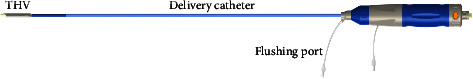
Venus-A plus valve delivery system. The flushing port is at 9 o'clock. THV = transcatheter heart valve.

The markers on the inflow of the 26 mm or 29 mm Venus series THV are in alignment with the nadirs of the THV sinuses. So it is possible to determine the positional relationship of the THV sinuses relative to the native coronary sinuses. The Venus-A plus THV is retrievable, but the delivery system is more inflexible than the first-generation Venus which cannot be retrieved. The Venus-A plus delivery catheter can't be rotated when the THV is at the aortic valve annulus. In addition, the delivery catheter only can be bent toward or away from the flushing port (Figure 5).

**Figure 6:**
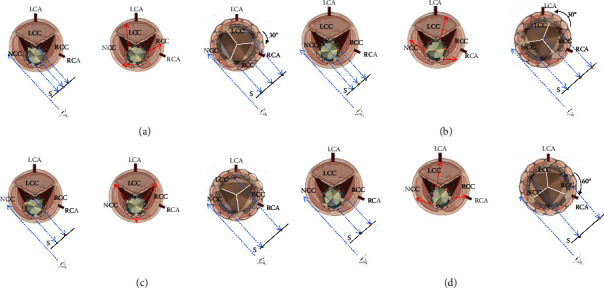
The three inflow markers on the THV are shown with green, red, and yellow dots. Each panel has three pictures. The first picture shows the position relationship between the THV and the aortic annulus when the THV crosses the aortic annulus. The red arrows in the second picture show the THV commissure moving direction when the THV is deployed. The third picture shows the position relationship between the THV and the aortic annulus after the THV has been deployed. The angulation between the neo-commissure and the native commissure will be 30° clockwise (a) or counter-clockwise (b) when the three markers are separated averagely in the projection with the right and left coronary cusps superimposing. When the centre marker is overlapped with the right one, the angulation between the neo-commissure and the native commissure will be 0° (c). When the centre marker is overlapped with the left one, the angulation between the neo-commissure and the native commissure will be 60°, leading to a total neo-commissural misalignment (d). S = screen; LCA = left coronary artery; RCA = right coronary artery; LCC = left coronary cusp; NCC = noncoronary cusp; RCC = right coronary cusp. The figure was created with biorender.com.

Deploying the self-expanding THV using a projection in which the right and left coronary cusps overlap has been a standard step during TAVR [8]. The aortic annulus and the inflow plane of the THV can be seen simultaneously, and the depth of THV implantation can be determined accurately in this projection. Furthermore, the noncoronary cusp will be isolated in this projection. The delivery catheter carrying the Venus-A plus valve will rotate by itself while being advanced from the femoral sheath to the aortic valve annulus clockwise or counter-clockwise by some degrees, which is different from one patient to another patient. The three markers on the THV will be distributed in three situations when the THV is advanced to the aortic annulus in the projection of the right and left coronary cusps overlapping. They are the three markers being separated averagely, with the centre marker being close to or overlapped with the left one or close to or overlapped with the right one. The relationships between the neo-commissure of the THV and the native commissure during the valve deployment are demonstrated in Figure 6. So, in our practice, we used the following strategy to deploy the Venus-A plus THV. In the projection with the right and left coronary cusps overlapping, we will deploy the THV directly if three markers are separated averagely or the centre marker is closed to or overlapped with the right one on the fluoroscopic image when the THV is at the aortic annulus. If the centre marker is closed to or overlapped with the left one on the fluoroscopic image, we will withdraw the delivery system to the descending aorta and rotate the delivery system 180°, then readvance the THV to the aortic annulus. The neo-commissural alignment or mild commissural misalignment was obtained in all five cases present in our study, although the THV rotated in the deploying phase by some degrees [2].

The Venus series valve is the first self-expanding valve which has three radiopaque markers providing a reference for deployment depth and commissure location. Recently, Evolut FX (Medtronic), the newest self-expanding valve in the Evolut series, is added three radiopaque golden markers in the valve inflow which are almost exact alignment with the THV commissures [9]. So the experience from the Venus-A plus valve mentioned in our study may be referred in the Evolut FX.

## 5. Limitations

The study sample is too small to allow for optimal statistical power. The possibilities of increasing the cerebral infarction risk are unknown because of the increased number of times that the THV crosses the aortic valve and because of the longer procedure time.

## 6. Conclusions

It is possible to obtain the neo-commissural alignment by controlling delivery catheter insertion orientation using the markers on the inflow of the Venus-A plus valve.

## Figures and Tables

**Table 1:** Baseline patient characteristics.

Case number	Gender	Age (years)	Aortic valve	Mean aortic valve gradient (mmHg)	Aortic blood flow velocity (m/s)	Venus-A plus THV (mm)
1	Male	85	Tricuspid	20	3	26
2	Female	66	Tricuspid	40	4.3	29
3	Male	73	Type I bicuspid	64	5.2	26
4	Male	53	Type I bicuspid	51	4.5	29
5	Male	69	Type I bicuspid	40	3.6	26

THV = transcatheter heart valve.

**Table 2:** Commissural angle of deviation before vs. post TAVR.

Case number	Commissural angle deviation before vs. post TAVR (°)	Neo-commissural misalignment	Neo-commissural alignment
1	20.7	Mild	
2	12.6		+
3	17.3	Mild	
4	7.3		+
5	3.7		+

## Data Availability

The clinical data used to support the findings of this study are available from the corresponding author upon request.
